# On Phytolacca Decandra

**Published:** 1881-04

**Authors:** W. C. Westerfield


### ARTICLE III.

### ON PHYTOLACCA DECANDRA. By W. C. WESTERFIELD, M.D.

In these days of new application of old remedies, and of restoration of the obsolete, I wish to introduce to the profession a long neglected yet highly virtuous member of our Materia Medica. There is but little history connected with Phytolacca Decandra, and what there is dates far back in our country's history in medical literature, with perhaps a few exceptions. We learn that at one time it held a high place as a remedial agent in the South, and especially in the Carolinas. Our oldest professional men will tell you that they have once heard of it as doing wonders in a curative way, but that it finally fell into disuse. Now, it is this neglect which I wish to inquire into, and if possible, explain as to how it came about.

In the year 1859, I wrote an article which was published in The *St. Louis Medical Journal* upon this subject, and which attracted quite an interest at the time; but the foot of war fell upon it, and it was tramped out of sight. At that time I wrote more from the experience of my father, who, in an active practice extending over a period of nearly forty years, had used it continuously. Since that time I have had recourse to it more than to any other remedy of its class, and can fully verify the opinion of it held by my predecessor, for it seldom fails to accomplish the object for which it is used, as for certainty of action it will take rank with the most unfailing of our remedies. The question then presses for an answer, Why has it fallen into disuse? A few years ago, and shortly after coming to Chicago, I desired to use the remedy in question, and procured it from one of our best druggists, but found that it did not have any impression upon the disease. I prescribed the fluid extract. I then had recourse to the freshly dug root from near the city, prepared a saturated tincture, feeling satisfied that now it would act as in former times, but to my surprise it did no such thing, which was as unexpected as if I had failed with quinine in an intermittent. I now determined to procure the roots from the same locality—where both my father and I had gotten it in former years—in the South, where, in protected places, the sun shone upon it from February to November. From this I carefully prepared a tincture, and again made an attack upon the same cases in which I had formerly failed, and with the great satisfaction of seeing it manifest its peculiar action within a few days, and shortly after, completely eradicate the diseases in question. I then ordered a quantity of the same roots and continue to use the remedy, with the satisfaction of seeing its old-time virtues still present.

Now, after this, and many subsequent proofs, I feel that the question as to its disuse has been solved, and that climate—not the remedy—is the cause of its failure in the hands of the physicians in the North. The perfumer must use the flowers from semi-tropical regions. Many of our vegetables deteriorate when transplanted from a warmer climate, and why not phytolacca decandra? In the Southern States this plant assumes gigantic proportions as compared with that grown in the North. With such diminution in growth, we may infer a diminished virtue, aside from the test applied to it.

The plant is too well known to enter into an elaborate description of it, but one thing I wish to mention, with all due deference and modesty, that which I have not seen mentioned elsewhere. My father and I recognized two varieties of this plant.

It may have been the conditions of growth, or the peculiarity of location—yet the two are so distinct as to merit mention at least—not botanically, but in their general appearance. One is a tall, tree-like plant—in some specimens twelve feet in height—flowers few, fruit small and sparse, main stalk and branches comparatively slender, and of a light green color. The other, and the one preferred, is more compact in general appearance, full, dense foliage of a dark-green color, fruit large and abundant, main stalk and larger branches of a deep, rich purple. The root, the part used, is thick and fleshy, abundant with starch and mucilaginous matter of a sweet mawkish taste, tardily but decidedly pungent to the mouth; a peculiarity, by the way, not found in the fluid extract nor in the root of this climate. It is best gathered in November, and must be carefully dried in the shade. Dampness is fatal to it, fermenting the starch, and for this reason it will not keep over until the following season, and that found in the drug stores is generally inert for that reason. The liquid after digestion, should never be evaporated except in vacuo.

The indications for its use are varied, fulfilling, as it does, the want so much felt in that class of remedies known as alteratives.

In obstructed or sluggish glandular action it may be used with excellent effect. This is especially true of all liver disorders. Also in all eruptive or desquamative skin diseases. I have seen some of the most obstinate and long standing of this latter class completely eradicated within a few weeks' time, after having resisted every known remedy in the hands of some of our best physicians. In rheumatism, too, just after the acute stage has been passed, and especially in the chronic form, it has done much service. In scrofulous diseases it may be used from the outset of the first symptoms, especially in the eruptive kind.

One peculiarity I must mention, which occurs after its use for eight or ten days, and that is, there appears an eruption upon the breast and back—sometimes on the face—about the size of millet seed. This, however, disappears in a short time, and with it, eruptions of skin diseases. This is often the process of cure, yet it will occur without this phenomenon, as it does not occur in all cases.

As corroborative of its effect upon glandular troubles, I will mention the fact that its effect upon irritation of the ovaries, with uterine complications, is most pronounced, especially where there is a tendency to hysteria. I have also seen, from an article by one of my father's former pupils, that he claims almost specific virtues in it, in threatened abscess of the mammary glands, in the beginning of lactation. I have had a similar experience, also in a case of irritation of the ovaries in a recent case which yielded within a week's time. In my father's practice a favorite combination was phyto. decan. and guaiac. In some cases, however, the latter was found to be too stimulating, and was replaced by pipsissewa, which proved to be an excellent combination. I now use it in connection with salicylate of soda, especially in preventing transition from acute to chronic. But, given alone, in as full doses as can be well borne, the phyto. decan. will be found to be an excellent remedy in all forms of subacute and chronic rheumatism. It has been recommended in syphilis, and in syphilo-dermata in connection with mercury, it has done efficient work, and seems to facilitate a cure in all forms where mercury is used.

In discolorizations of the skin, termed blotches, in deposits of yellow "bilious" pigment, and in acneiform pimply skins, it has proven quite efficacious. The manner of administering it is to give a teaspoonful three times a day, gradually increasing to two teaspoonfuls, in a little water. It sometimes produces nausea, if given in full doses at first. In this case the reduction of the dose, or the addition of tr. gent. com., but sometimes nothing less than a cessation for a time, will suffice. Yet, with some, large doses are well borne. A gentleman recently took half an ounce, four times a day, for removing pimples from his forehead, with the effect of a free purgation only, and the removal of the eruption within a week.

I could mention many cases of cure and of beneficial results, but will not at this time. What is here written is for the purpose of enlisting others in a trial of the remedy. There are given here only a few hints as to its field of usefulness, hoping that others more competent may find, as I have, that in phytolacca decandra we have a remedy which may be depended upon.

